# Risk Factors for Cataracts in Patients with Diabetes Mellitus

**DOI:** 10.3390/jcm13237005

**Published:** 2024-11-21

**Authors:** Adriana Ivanescu, Simona Popescu, Laura Gaita, Oana Albai, Adina Braha, Romulus Timar

**Affiliations:** 1 Department of Second Internal Medicine Diabetes, Nutrition, Metabolic Diseases, and Systemic Rheumatology, “Victor Babes” University of Medicine and Pharmacy, 300041 Timisoara, Romania; adriana.ivanescu@umft.ro (A.I.); gaita.laura@umft.ro (L.G.); albai.oana@umft.ro (O.A.); braha.adina@umft.ro (A.B.); timar.romulus@umft.ro (R.T.); 2 Opticlass Ophtalmology Clinic, 300012 Timisoara, Romania; 3 Doctoral School, “Victor Babes” University of Medicine and Pharmacy, 300041 Timisoara, Romania; 4 Department of Diabetes, Nutrition, and Metabolic Diseases Clinic, “Pius Brînzeu” Emergency Clinical County University Hospital, 300723 Timisoara, Romania

**Keywords:** diabetes mellitus, cataracts, cataract subtypes, risk factors

## Abstract

**Background:** Diabetes mellitus (DM) is one of the most impactful health problems worldwide. It affects ocular health in multiple ways and is one of the leading causes of vision loss. Our study aimed to evaluate the most important systemic risk factors related to the occurrence of cataracts in patients with DM. **Method:** This study evaluated a final number of 319 participants who were previously diagnosed with DM. For all patients, we retrieved data regarding DM status, metabolic control, demographic and anthropometric indices, and generally associated comorbidities from their medical charts. A comprehensive eye examination was performed on all patients. **Results:** The main studied risk factors were hypertension, cardiovascular disease (CVD), chronic kidney disease (CKD), diabetic polyneuropathy (DPN), dyslipidemia, and hepatic steatosis, which were present among the entire population. Hypertension (67.6%), DPN (53.3%), and dyslipidemia (46.6%) were highly prevalent in the cataract subgroup, and CKD (*p* < 0.001) and DPN (*p* = 0.019) were found to be predictive factors for the probability of cataract occurrence. Ophthalmologic evaluation was used to assess the presence of ocular complications, such as diabetic retinopathy (DR) and diabetic maculopathy. DR reached statistically significant values in the occurence of cataracts. Patients’ age and DM-related factors, such as disease duration (*p* < 0.001) and HbA1c values (*p* = 0.029), significantly increased the risk of cataracts. Smoking was self-reported by 24.8% of the patients, with a significant impact on the occurrence of cataracts (*p* = 0.04). **Conclusions:** Patients with DM who exhibit a longer disease duration and poor glycemic control in conjunction with systemic comorbidities present a higher risk of developing cataracts; consequently, a strict therapeutic approach regarding these risk factors is needed.

## 1. Introduction

Diabetes mellitus (DM) is a chronic metabolic disease that has rapidly expanded in both developed and developing countries and is anticipated to reach epidemic proportions in the near future. DM is a serious health problem that leads to significant morbidity due to specific microvascular complications, such as retinopathy, nephropathy, and neuropathy, as well as macrovascular complications such as ischemic heart disease and peripheral vasculopathy. Aside from diabetic retinopathy (DR), DM can also cause several other ocular complications, including cataracts, diabetic papillopathy, and glaucoma [1].

One of the most frequent and severe ocular pathologies is cataracts, which alter the crystalline lens and determine both short- and long-term eyesight impairment [2]. Clinical, epidemiological, and basic studies have linked diabetes to cataract development. As the global population of individuals with type 1 (T1DM) and type 2 diabetes mellitus (T2DM) continues to grow, the prevalence of cataracts in the diabetic population is also increasing [3].

The proportion of blindness attributable to cataracts among all eye diseases varies significantly, ranging from 5% in developed countries to 50% or more in poorer and/or remote regions [4]. Cataracts are one of the leading causes of vision loss worldwide, especially among the diabetic population. They occur two to five times more frequently in patients with DM and this frequency can increase by 15 to 25 times in individuals under 40 years of age [5].

A cataract represents the opacification that forms in the translucent lens of the eye, varying from partial to complete. This diminishes the clarity of the lens and leads to reduced visual acuity. It also impedes ophthalmologists’ ability to examine the posterior segment of the eye and can make certain treatments impossible, such as retinal photocoagulation [6,7].

The primary mechanisms contributing to cataract formation in patients with diabetes are the polyol pathway and oxidative stress. In the polyol pathway, aldose reductase (AR) catalyzes the conversion of glucose to sorbitol, a process directly associated with the onset of diabetic cataracts. Extensive research has emphasized the pivotal role of the AR pathway as a key initiator under these conditions. Sorbitol accumulation within cells leads to osmotic disturbances, causing swelling (hydropic changes) in the lens fibers, ultimately degenerating and forming cataracts [8,9]. Furthermore, studies have shown that osmotic stress in the lens resulting from sorbitol accumulation induces apoptosis in the lens epithelial cells (LECs), contributing to cataract development [10,11,12]. Osmotic stress is critical for the rapid formation of cataracts in young patients with T1DM, primarily because of the significant swelling of the cortical lens fibers. This accelerated lens damage highlights patients’ vulnerability to early cataract development [13,14]. The lens polyol pathway is the primary mediator of diabetes-induced oxidative stress. This pathway contributes to osmotic imbalance and promotes oxidative damage, which are key factors in the development of cataracts in patients with diabetes [15]. Osmotic stress resulting from sorbitol accumulation triggers endoplasmic reticulum (ER) stress, the central protein synthesis site producing free radicals. Additionally, fluctuating glucose levels can induce ER stress by activating the unfolded protein response, which generates reactive oxygen species (ROS). ROS contribute to oxidative stress, damage lens fibers, and promote cataract development in diabetic patients [16,17].

There are three main types of cataracts: cortical (CCs), nuclear sclerotic (NS), and posterior subcapsular (PSCs). Cataract progression can differ based on its type and factors, such as overall health and the environment [18,19]. The impact of cataracts on vision differs according to the location of the opacity, leading to varying symptoms in accordance with each type. The intensity of symptoms, such as decreased visual acuity, reduced contrast sensitivity, glare, and impaired color vision, depends on the stage of cataract development [20]. Each type of cataract can occur independently, although they co-occur more commonly [17]. The current literature indicates a higher prevalence of the PSC and CC subtypes in patients with diabetes [21], whereas NS cataracts are more frequent among patients without diabetes [22].

Cataracts, in conjunction with diabetes, pose a significant health and economic burden, especially in developing countries where diabetes treatment is inadequate and cataract surgery is often inaccessible. Considering these aspects, healthcare providers must identify vision-threatening pathologies such as cataracts in individuals with DM as early as possible, as well as the possible risk factors involved in its development, to achieve optimal management of ocular health in the diabetic population.

Despite the well-known importance of this pathology, there is limited literature on the prevalence and risk factors involved in cataract occurrence in patients with DM [7,23,24]. The conventional risk factors for DM include poor metabolic control and elevated HbA1c levels. However, most diabetes patients present with various associated systemic diseases that affect cataract occurrence [25,26,27].

To obtain successful treatment outcomes in the diabetic population with cataracts, the management of these patients must involve a multidisciplinary approach that targets strict control of general risk factors, as well as individualized ophthalmologic treatment to reduce visual symptoms, enhance visual function, attain the desired refractive state, and improve mental well-being [28,29].

Considering the significant impact of cataracts on the quality of life of patients with diabetes, this study aimed to investigate the most important risk factors involved in cataract development to sensitize physicians to the importance of screening and early detection of cataracts and overall health among these patients.

## 2. Materials and Methods

### 2.1. Study Design and Population

This non-interventional, cross-sectional study was conducted at the Outpatient Diabetes Care Center of Pius Brinzeu County Emergency Hospital in Timisoara between 16 July 2024 and 16 September 2024. Of the 523 adult patients (over 18 years old) who visited their prescheduled visits at the diabetes center, we enrolled a final number of 319 participants with DM (167 females, 152 males). Of the total number of patients, 96 declined to participate in this study and 108 did not meet the study’s eligibility criteria. The exclusion criteria were severe cognitive impairment and psychiatric or neurological pathologies that prevented patients providing informed consent, institutionalized patients, and other medical disorders that required hospitalization throughout the study. In addition, patients with other cataract subtypes, such as congenital or traumatic glaucoma, extensive corneal opacities, and other significant ocular surface diseases that impede proper anterior segment evaluation, were excluded. None of the participants were involved in the development of this study, and informed consent was obtained from the entire research group. This study was approved by the Ethics Committee of the Emergency County Hospital, “Pius Brinzeu” Timisoara (approval no. 473/15 July 2024), and was conducted in accordance with the Declaration of Helsinki (2013 version). The selected patients were aged > 18 years and had been previously diagnosed with DM. In our research group, participants had a median DM duration of 10 years [6,14] and a median age of 68 [63,76]. All the patients were evaluated by their physicians and subjected to a comprehensive eye examination performed by an ophthalmologist. The study protocol included the evaluation of anthropometric and demographic characteristics, DM profile and metabolic control, systemic comorbidities, diabetes-related ocular complications, and smoking status.

### 2.2. Data Collection and Medical Assessment

Demographic and anthropometric data such as weight and height were retrieved from the medical records of each patient. Body mass index (BMI) was calculated using a metric system. DM diagnosis had been previously established using one or more of the following criteria: fasting plasma glucose level more than 7.0 mmol/L (126 mg/dL), a 2 h post-load plasma glucose level > 11.1 mmol/L (200 mg/dL), or an HbA1c level > 6.5% (48 mmol/mol).

Hypertension was diagnosed with a systolic blood pressure ≥ 140 mmHg and/or diastolic blood pressure ≥ 90 mmHg. Cardiovascular disease (CVD) referred to coronary heart disease, stroke, or peripheral arterial disease and was noted according to the patient’s medical history.

Nerve conduction velocity (NCV) and the Michigan Neuropathy Screening Instrument (MNSI) were used to diagnose diabetic polyneuropathy (DPN), defined as NCV values < 40 m/s and an MNSI clinical overall score of > 9.5.

Creatinine-based estimates of the glomerular filtration rate (eGFR) and albumin/creatinine ratio (ACR) were evaluated, and chronic kidney disease (CKD) diagnoses were formulated accordingly. Simultaneously, the presence of dyslipidemia was reported according to the lipid profile. The above results and hepatic steatosis diagnoses were retrieved from patients’ medical charts. Smokers were self-reported and only active smokers were considered.

A board-certified ophthalmologist performed a complete slit-lamp biomicroscopy (Topcon SL-D2 biomicroscope, Tokyo, Japan) with pupil dilation on all participants. Anterior segment evaluation included examination of the cornea, iris, and crystalline lens, focusing mainly on cataract identification. Cataract diagnosis was formulated according to the Lens Opacity Classification System (LOCS) III by evaluating the presence and type of lens opacities, focusing on the three main cataract subtypes: CCs, NS, and PSCs. CCs were diagnosed based on spoke-like or wedge-shaped opacities at the periphery of the crystalline lens. NS cataracts were diagnosed as yellow, hardened lens nuclei, whereas PSCs were diagnosed when opacified plaques or granular opacities were identified in the posterior cortex. Posterior pole examination for each patient identified DR and diabetic maculopathy; however, this study did not evaluate the severity degree of these ocular pathologies. Intraocular pressure (IOP) was also measured for all participants after prior application of anesthetic drops and fluorescein dye using a Perkins handheld applanation tonometer (Perkins Mk3 tonometer, Haag-Streit, Koniz, Switzerland), with one measurement performed for each eye [30].

### 2.3. Statistical Analysis

Statistical analysis was performed using MedCalc^®^ Statistical Software version 23.0.5 (MedCalc Software Ltd., Ostend, Belgium; https://www.medcalc.org; accessed on 30 July 2024). We applied the Shapiro–Wilk test to check the distribution of the variables. Variables with normal distribution were described as mean, standard deviation, and variables with non-normal distribution were described as median and minimum–maximum or interquartile range (IQR) intervals. The variables were presented as absolute values and percentages. The Mann–Whitney U test was used to compare non-parametric variables between the two groups, and the unpaired U test was used for parametric variables. The frequencies of the studied comorbidities were compared using the Chi-squared test. To evaluate the impact of the analyzed factors (HbA1c level, age, duration of diabetes, and comorbidities) on the development of cataracts, we applied multiple logistic regression analysis stepwise. The results are presented as odds ratios (ORs) with confidence intervals (CIs), indicating the strength of the association. Subsequently, we evaluated the diagnostic power of the studied clinical parameters to identify patients with cataracts by analyzing the receiver operating characteristic (ROC) curves and calculated the area under the curve (AUC), sensitivity, and specificity for age, diabetes duration, and HbA1c level as potential predictors of cataracts. We then constructed a precision-recall curve, a plot of the precision (positive predictive value, *y* axis) against the recall (sensitivity, *x* axis), for different thresholds of age and diabetes duration, reporting the difference between the areas under the precision-recall curve and the bootstrap confidence interval. We concluded that the areas were significantly different if they did not include zero. In addition, we reported the F1 max to test the accuracy of the results for the association criteria. We evaluated the risk factors associated with different cataract subtypes while controlling for confounding variables using the Cochran–Mantel–Haenszel odds ratio analysis. The level of statistical significance was set at *p* < 0.05.

## 3. Results

The entire study population included 319 patients, with a slight female predominance (52.3%), a median age of 68 (IQR: 61–76) years, and a median disease duration of 10 years (IQR: 6–14) years. Both males and females exhibited similar levels of metabolic control, as indicated by the median HbA1c levels (7.3% in men, 7.5% in women, *p* = 0.710). The general characteristics of the study population are summarized in Table 1.

**Figure 1:**
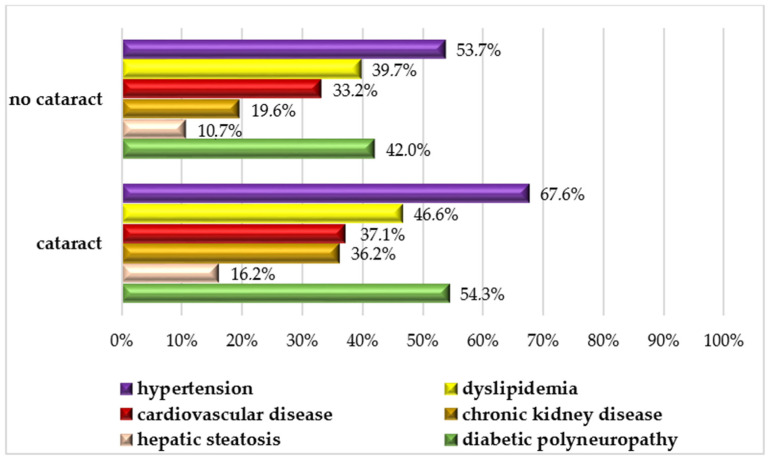
Prevalence of associated comorbidities across the entire study population, stratified by cataract status.

CKD was significantly more prevalent in men (33.6%) compared to women (17.4%, *p* < 0.001), as was hepatic steatosis (18.4% in men vs. 7.2% in women, *p* = 0.002). The prevalence of DR, hypertension, CVD, and dyslipidemia did not differ significantly between men and women, as can be seen in Figure 1.

Regarding the ophthalmological findings, 65.5% of the population had DR, 42.3% had diabetic maculopathy, and 32.9% had cataracts, with a similar prevalence across sexes.

When the study population was compared based on the presence or absence of cataracts, several statistically significant differences were observed (Table 2). Patients with cataracts were older, had a longer duration of diabetes, poorer metabolic control, and were more likely to be smokers. Additionally, comorbidities such as hypertension, CKD, DPN, and DR were more prevalent in diabetic patients with cataracts.

A subgroup analysis of cataract patients was performed to evaluate potential differences, as shown in Table 3. Female patients with cataracts had significantly higher HbA1c values (median 8%) than men (median 7.5%, *p* = 0.010). Most patients were overweight, with a median BMI of 28.6 kg/m^2^. The most common general comorbidities were hypertension (67.6%), DPN (53.3%) and dyslipidemia (46.6%), with no significant sex differences observed in the prevalence of these conditions. Smoking was self-reported by 24.8% of the patients. No significant differences were observed in the prevalence of DR or diabetic maculopathy. In the cataract subgroup, NS cataracts were significantly more frequent in male patients (*p* = 0.002).

**Figure 2:**
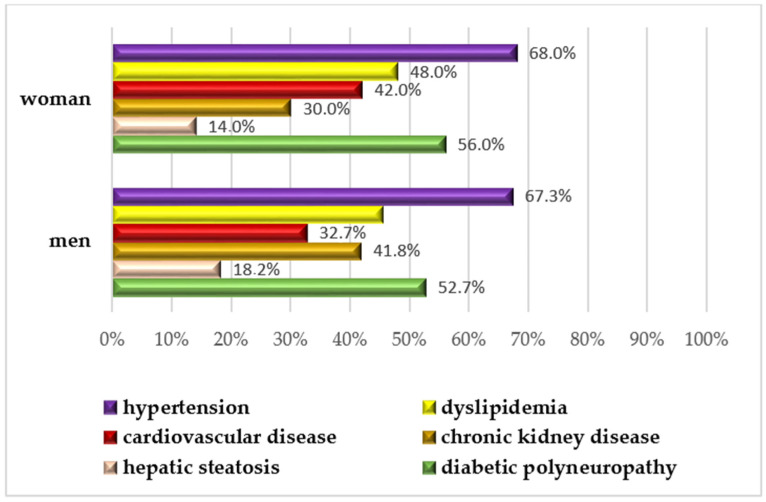
Prevalence of associated comorbidities across cataract patients, stratified by gender.

The most common general comorbidities were hypertension (67.6%), DPN (53.3%), and dyslipidemia (46.6%), with no significant sex differences observed in the prevalence of these conditions, as seen in Figure 2.

**Figure 3:**
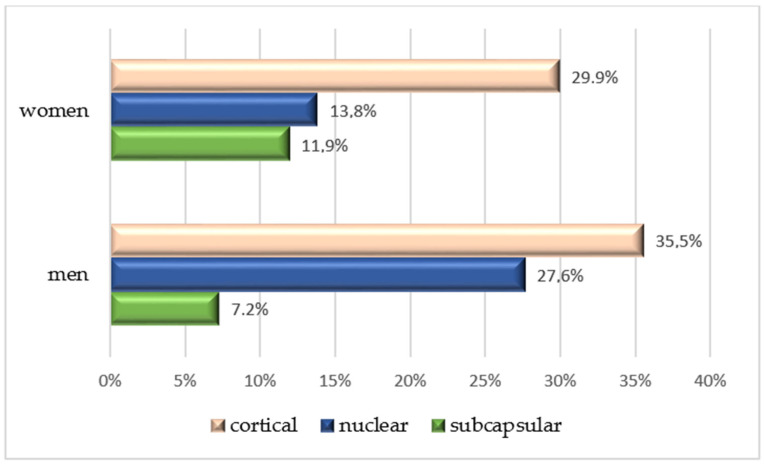
Prevalence of cataract subtypes, stratified by gender.

Smoking was self-reported by 24.8% of the patients. No significant differences were observed in the prevalence of DR or diabetic maculopathy. In the cataract subgroup, NS cataracts were significantly more frequent in male patients (*p* = 0.002), as shown in Figure 3.

Multiple logistic regression was applied to assess the adjusted impact of HbA1c levels, age, diabetes duration, and various comorbidities on cataract development. As shown in Table 4, the risk of cataracts significantly increased with age, disease duration, HbA1c levels, and the presence of CKD and diabetic DPN.

**Figure 4:**
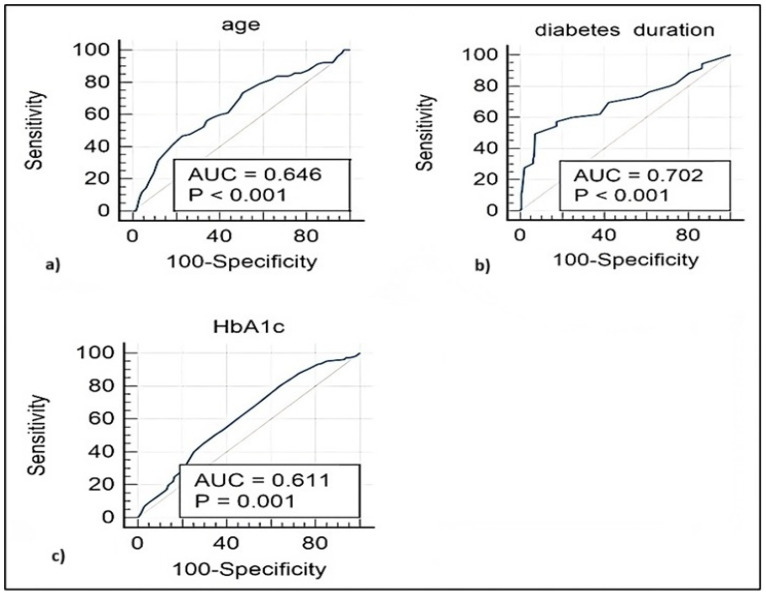
Receiver operating characteristic curve analysis for evaluating the performance of (**a**) age, (**b**) diabetes duration, and (**c**) HbA1c in discriminating cataract development in diabetic patients.

We further assessed the diagnostic value of several clinical parameters in identifying patients with cataracts across the entire study population by performing an ROC curve analysis. As summarized in Table 5 and Figure 4, disease duration had the highest discriminative capacity for cataracts, with an optimal cutoff point of 14 years and an area under the curve (AUC), sensitivity, and specificity of 0.702, 49.5%, and 92.9%, respectively. According to the ROC curve analysis, age, and HbA1c levels showed borderline discriminative capacities (Table 5).

**Figure 5:**
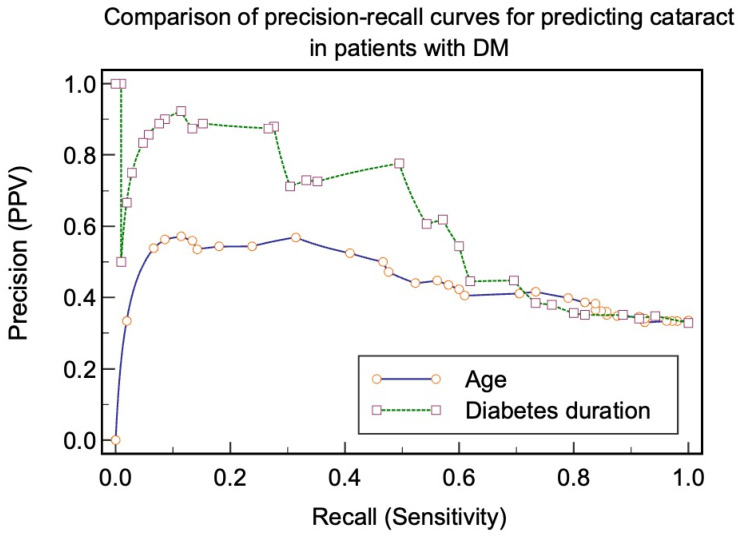
Comparison of precision-recall curves of age and diabetes duration as predictors of the presence of all types of cataracts.

Diabetes duration of >14 years predicted the presence of cataracts with a recall (sensitivity) of 0.49, precision (PPV) of 0.77, and F1 max of 0.60. Age of >66 years predicted the presence of cataracts with a recall (sensitivity) of 0.73, precision (PPV) of 0.41, F1 max of 0.53, and AUROC of 0.455. The comparison of paired precision-recall curves showed a difference of 0.16 between areas, a 95% CI 0.36–0.55 for age, and 0.52–0.71 for diabetes duration (Figure 5).

Table 6 outlines the risk factors associated with cataract subtypes, focusing on the HbA1c levels. Patients with poorer metabolic control (HbA1c ≥ 7%) had increased odds of developing NS cataracts if they had CVD (OR = 5.62, 95% CI: 1.27–24.79, *p* = 0.01), dyslipidemia (OR = 5.48, 95% CI: 1.24–24.03, *p* = 0.01), CKD (OR = 4.34, 95% CI: 1.13–16.61, *p* = 0.02), hepatic steatosis (OR = 5.40, 95% CI: 1.22–23.93, *p* = 0.01), DPN (OR = 4.76, 95% CI: 1.22–18.45, *p* = 0.01), or if they were smokers (OR= 5.17, 95% CI: 1.18–22.62, *p* = 0.01). The odds of patients with poorer metabolic control developing CCs were even higher in the presence of CVD (OR = 6.19, 95% CI: 1.40–27.32, *p* = 0.006), hepatic steatosis (OR = 5.78, 95% CI: 1.29–25.91, *p* = 0.01), dyslipidemia (OR = 5.78, 95% CI: 1.30–25.63, *p* = 0.01), CKD (OR = 4.30, 95% CI: 1.11–16.58, *p* = 0.02), DPN (OR = 5.21, 95% CI: 1.32–20.47, *p* = 0.01), or if they were smokers (OR = 5.55, 95% CI: 1.26–24.43, *p* = 0.01).

## 4. Discussion

This study aimed to evaluate the most important risk factors for cataract occurrence and development in patients with diabetes. Of all the study participants, 32.9% presented with cataracts, results that are similar to those in recent studies. An important study conducted by Memon et al. showed that among individuals under 40, 33.3% of those with diabetes developed cataracts. In the 40–59 age group, the prevalence was 41% of people with diabetes. Similarly, among people with diabetes over the age of 60, 47% develop cataracts [7]. In a retrospective observational study of 56,510 patients with diabetes, Becker et al. obtained similar results [31]. This study included an elderly diabetic population with a median age of 68. Our results showed that cataract occurrence increased with age (*p* < 0.001), which is consistent with the current literature. Considering the high prevalence of cataracts among younger patients with diabetes, we believe that our study population should be extended to a younger demographic group in future analysis [5,7,32].

The evaluated group included 319 diabetic patients, with a slight female predominance (52.3%). In our cataract subgroup (105 participants), we observed a similar sex distribution (47.6% females). In our study, sex was not statistically related to cataract occurrence in general; however, male patients mostly presented with NS cataracts (*p* = 0.002). The current literature generally states that female diabetic patients are more prone to developing cataracts, especially at a younger age [31,32]. In contrast, other studies have reported a positive correlation between cataracts and male participants [33].

As stated above, our study focused on evaluating the most frequent cataract subtypes in relation to the systemic risk factors. Of the three main cataract subtypes, NS cataracts and PSCs, NS cataracts are the most frequent in the general population [19]. Simultaneously, the Beaver Dam Eye Study cohort found a significant association between DM, CCs, and PSC subtypes [34]. Considering these results, an important aspect of our study is that we included patients who presented with multiple types of associated lens opacities, with CCs being predominant in the cataract group (104 of 105 patients diagnosed with cataracts), followed by NS cataracts and PSCs.

Regarding DM, patients presented with significant disease lengths, with a median duration of 14 years and poor glycemic control reflected by a median HbA1c value of 7.7%. Both factors strongly predicted cataract occurrence (DM duration, *p* < 0.001; HbA1c level, *p* = 0.029). Furthermore, the NS cataract and CC subtypes were significantly associated with inadequate glycemic control. The negative effects of DM duration and uncontrolled DM have been consistently evaluated and confirmed by a number of studies in the current literature [31,35,36].

Our study population consisted mainly of overweight diabetic patients, reflected by a mean BMI (kg/m^2^) of 28.6% in the cataract group. However, no statistically significant results were found regarding the effect of BMI on cataract occurrence or subtype. In contrast, in the current literature, some studies have linked higher BMI to the PSC subtype [37].

Cataracts progress more rapidly in smokers [38]. In our study, smoking was self-reported by 24.8% of participants in the cataract group, and results have shown a significant association between NS cataract and CC occurrence (*p* < 0.001). The data available in the current literature mostly link smoking to the development of NS cataracts; however, little evidence exists regarding PSCs or CCs [39]. Also, it must be noted that we took into consideration only active smokers and information regarding the number of cigarettes or the duration of this habit was unavailable, data that we consider of interest for future research as there are studies that link the risk of cataract development to the number of cigarettes smoked [40].

Our study population mainly represented diabetic patients with poor glycemic control, ocular diabetes complications such as DR, or diabetic maculopathy; so, as expected, more patients presented with a combination of these pathologies. DR and diabetic maculopathy have a significant impact on ocular health because they are vision-threatening pathologies. At the same time, DR has also been stated to be involved in cataract development and progression. In agreement with these data, DR was significantly associated with cataracts (*p* = 0.04) [41]. IOP was also measured during the ophthalmologic evaluation, but was within the normal range among the studied population and did not reach statistical significance.

With the global increase in the aging population [42], the concept of multimorbidity has gained growing interest among healthcare providers [43], particularly for diabetic patients who are at a higher risk of developing multiple concurrent pathologies [44]. In our study population, most participants presented with at least one comorbidity besides DM. Our primary interest was to identify major systemic conditions, including CKD, CVD, hypertension, hepatic steatosis, DPN, and dyslipidemia, which are more prevalent in older patients and have a notable impact on diabetes-related ophthalmologic complications such as vision-threatening cataracts. As previously mentioned, pathologies were highly prevalent in the study group. More importantly, these comorbidities were notably higher in the subpopulation of patients with cataracts. CKD and hepatic steatosis were the most significant risk factors. These results are somewhat expected as these pathologies share similar pathogenesis mechanisms with cataracts, specifically the polyol pathway and oxidative stress [45].

In our study group, we observed a higher CKD prevalence among the male participants (33.6% vs. 17.4%), a possible explanation for which could the cross-sectional nature of our study. Patients were included in order of their attendance at prescheduled visits to the Diabetes Care Center. The current literature mainly states a link between female sex and CKD prevalence [46], while other studies relate male sex to being more affected by CKD [47]. Another aspect of our population is that hepatic steatosis was significantly more prevalent in the male participants (*p* = 0.002), and hepatic steatosis is known to be linked to CKD [48]. It is also known that CKD prevalence is higher among smokers [49,50] and in our study, smoking was more prevalent in the male participants (32 male smokers vs. 25 female smokers). Therefore, all the above data could explain the higher CKD prevalence among men in our study group.

The concept of empowerment has recently gained increasing interest among healthcare providers because it aims to promote a better relationship between patients and physicians [51]. In Romania, in general, monitoring a diabetic patient implies check-ups for at least 3 months, depending on the DM subtype and health status, and at least one annual screening for chronic complications. Each visit implies re-educating each patient regarding diabetes management and possible complications. Furthermore, healthcare specialists in diabetes, nutrition, and metabolic diseases organize diabetes awareness campaigns to increase health education among the general population. However, the adherence of diabetic patients to medical treatment regimens and dietary recommendations is known to be inadequate, especially in developing countries such as Romania, making the empowerment concept a difficult goal to obtain [42].

With regard to patient ocular health education, cataract symptoms should be emphasized. Older patients benefit significantly from improved quality of life if cataracts are addressed and surgically treated. Moreover, some studies have stated that patients with diabetes have better metabolic control after cataract surgery, making timely ophthalmologic intervention even more important [52].

The strengths of our study include the significant study population, which reliably reflects the general health status of diabetic patients with cataracts on our side of the country. However, this study has a few limitations, such as the single-center nature of the research. Therefore, results involving ophthalmologic findings or therapeutic approaches might vary. Even if multivariate logistic regression is applied, a few variables such as genetic and environmental factors were not considered. Consequently, longitudinal multi-center research should be considered in the future. By outlining the present results, we aim to raise awareness among healthcare providers of the fact that cataract development in diabetic patients is not only significantly influenced by conventional factors such as diabetes duration or glycemic control, but also by many systemic pathologies that should be strictly taken into consideration in the management of patients to improve quality of life and maintain proper ocular health.

## 5. Conclusions

Our findings are consistent with those in the current literature, emphasizing that poor glycemic control and extended DM duration are the most critical risk factors for cataract development in patients with diabetes. Patients with DM are known to present with multiple associated pathologies such as hypertension, CVD, CKD, DPN, hepatic steatosis, and dyslipidemia. The present study identified CKD and DPN as the most influential comorbidities on cataract occurrence and development, whereas hepatic steatosis was linked to NS cataracts. While maintaining optimal glycemic control remains a cornerstone strategy for preventing ophthalmological complications in patients with DM, early detection and management of all risk factors prevents the occurrence of vision-threatening cataracts and aids healthcare providers in obtaining better patient-related outcomes.

## Figures and Tables

**Table 1:** General characteristics of the entire study population.

Variable	Overall (*n* = 319)	Men (*n* = 152)	Women (*n* = 167)	*p*-Value
Age (years)	68 (61, 76)	68 ± 9.4	68.4 ± 9.6	0.588 *^t^*
DM duration (years)	10 (6, 14)	10 (7, 14)	9 (6, 14)	0.355 *^U^*
Weight (kg)	82 (72.3, 90)	83 (75, 92)	80 (72, 89)	**0.029** *^U^*
BMI (kg/m^2^)	27.9 (24.8, 30.9)	26 (23.1, 29)	29.4 (26.6, 32.6)	**<0.001** *^U^*
Abdominal circumference (cm)	92.9 ± 13.6	94.8 ± 13.1	91.2 ± 13.9	**0.011** *^t^*
Glycemia (mg/dL)	178 (156, 195)	178 (154, 195)	178 (156, 195)	0.681 *^U^*
HbA1c (%)	7.5 (6.8; 8)	7.30 (6.80, 8.00)	7.50 (6.70, 8.05)	0.710 *^U^*
Smoking *n* (%)	57 (17.9)	32 (21.1)	25 (15)	0.193 ^χ2^
Hypertension *n* (%)	186 (58.3)	91 (59.8)	95 (56.9)	0.590 ^χ2^
Dyslipidemia *n* (%)	134 (42)	65 (42.8)	69 (41.3)	0.794 ^χ2^
CVD *n* (%)	110 (34.5)	55 (36.2)	55 (33)	0.542 ^χ2^
CKD *n* (%)	80 (25.1)	51 (33.6)	29 (17.4)	**<0.001** ^χ2^
Hepatic steatosis *n* (%)	40 (12.5)	28 (18.4)	12 (7.20)	**0.002** ^χ2^
DPN *n* (%)	147 (46.1)	70 (46.1)	77 (46.1)	0.993 ^χ2^
IOP right eye (mmHg)	15 (13, 17.)	16 (13.7, 17)	15 (13, 18)	0.831 *^U^*
IOP left eye (mmHg)	14 (14, 18)	16 (14, 18)	16 (14, 18)	0.921 *^U^*
Mean IOP (mmHg)	15.5 (13.5, 17.5)	15.75 (14, 17.5)	15.5 (13.5, 17.5)	0.853 *^U^*
DR *n* (%)	209 (65.5)	100 (65.7)	109 (65.2)	0.922 ^χ2^
Diabetic maculopathy *n* (%)	135 (42.3)	65 (42.7)	70 (41.9)	0.878 ^χ2^
Cataract *n* (%)	105 (32.9)	55 (36.1)	50 (29.9)	0.234 ^χ2^

*^t^* Independent sample *t*-test, *^U^* Mann–Whitney U test, and ^χ2^ Chi-square test. Data are presented as mean ± standard deviation, median (IQR), or percentage (*n*, %), as appropriate. DM, diabetes mellitus; BMI, body mass index; HbA1c, hemoglobin A1c; CVD, cardiovascular disease; CKD, chronic kidney disease; DPN, diabetic polyneuropathy; IOP, intraocular pressure. Statistically significant differences, indicated by a *p*-value < 0.05, are highlighted in bold.

**Table 2:** Comparison of studied parameters by the presence of cataracts.

Variable	Cataract + (*n* = 105)	Cataract − (*n* = 214)	*p*-Value
Age (years)	71.2 ± 8.9	66.7 ± 9.5	**<0.001** *^t^*
Females *n* (%)	50 (47.6)	117 (54.7)	0.236
DM duration (years)	14 (8, 20)	9 (5, 11)	**<0.001** *^U^*
Weight (kg)	82 (75, 92)	80 (72, 90)	0.189 *^U^*
BMI (kg/m^2^)	28.6 (25.1, 32.6)	27.8 (24.4, 30.6)	0.053 *^U^*
Abdominal circumference (cm)	94.2 ± 14.4	92.4 ± 13.2	0.269 *^t^*
Glycemia (mg/dL)	184 (162, 202)	177 (153, 190)	**0.002** *^U^*
HbA1c (%)	7.7 (7, 8.2)	7.2 (6.5, 7.9)	**0.001** *^U^*
Smoking *n* (%)	26 (24.8)	31 (14.5)	**0.040** ^χ2^
Hypertension *n* (%)	71 (67.6)	115 (53.7)	**0.018** ^χ2^
Dyslipidemia *n* (%)	49 (46.6)	85 (39.7)	0.237 ^χ2^
CVD *n* (%)	39 (37.1)	71 (33.2)	0.484 ^χ2^
CKD *n* (%)	38 (36.2)	42 (19.6)	**0.001** ^χ2^
Hepatic steatosis *n* (%)	17 (16.2)	23 (10.7)	0.168 ^χ2^
DPN *n* (%)	57 (54.3)	90 (42)	**0.039** ^χ2^
IOP right eye (mmHg)	16 (14, 17)	15 (13, 17.5)	0.160 *^U^*
IOP left eye (mmHg)	16 (14, 18)	15 (13, 17.8)	0.061 *^U^*
Mean IOP (mmHg)	16 (14.5, 17.5)	15.5 (13, 17.5)	0.064 *^U^*
DR *n* (%)	77 (73.3)	132 (61.7)	**0.040** ^χ2^
Diabetic maculopathy *n* (%)	47 (44.8)	88 (41.1)	0.536 ^χ2^

*^t^* Independent sample *t*-test, *^U^* Mann–Whitney U test, and ^χ2^ Chi-square test. Data are presented as mean ± standard deviation, median (IQR), or percentage (*n*, %), as appropriate. DM, diabetes mellitus; BMI, body mass index; HbA1c, hemoglobin A1c; CVD, cardiovascular disease; CKD, chronic kidney disease; DPN, diabetic polyneuropathy; IOP, intraocular pressure. Statistically significant differences, indicated by a *p*-value < 0.05, are highlighted in bold.

**Table 3:** General characteristics of the cataract subgroup.

Variable	Men (*n* = 55)	Women (*n* = 50)	*p*-Value
Age (years)	71.9 ± 8.3	70.3 ± 9.6	0.350 *^t^*
DM duration (years)	14 (9, 20)	15 (7.2, 18.7)	0.908 *^U^*
Weight (kg)	82 (77, 90)	82.5 (75, 92)	0.669 *^U^*
BMI (kg/m^2^)	26.4 (24.6, 29.8)	30.8 (27.4, 34.2)	**<0.001** *^U^*
Abdominal circumference (cm)	94.7 ± 13.9	93.6 ± 15	0.691 *^t^*
Glycemia (mg/dL)	184 (156, 197)	184.5 (162.3, 211)	0.388 *^U^*
HbA1c (%)	7.5 (6.8, 8)	8 (7.3, 8.6)	**0.010** *^U^*
Smoking *n* (%)	15 (27.3)	11 (22)	0.411 ^χ2^
Hypertension *n* (%)	37 (67.3)	34 (68)	0.937 ^χ2^
Dyslipidemia *n* (%)	25 (45.5)	24 (48)	0.794 ^χ2^
CVD *n* (%)	18 (32.7)	21 (42)	0.326 ^χ2^
CKD *n* (%)	23 (41.8)	15 (30)	0.208 ^χ2^
DPN *n* (%)	29 (52.7)	28 (56)	0.737 ^χ2^
Hepatic steatosis	10 (18.2)	7 (14)	0.561 ^χ2^
IOP right eye (mmHg)	16 (14, 17)	16 (14, 18)	0.328 *^U^*
IOP left eye (mmHg)	15 (14, 17.5)	16 (14, 18)	**0.012** *^U^*
Mean IOP (mmHg)	15.5 (14.5, 17)	16.3 (15, 18)	0.080 *^U^*
DR *n* (%)	40 (72.7)	37 (74)	0.883 ^χ2^
Diabetic maculopathy *n* (%)	26 (47.2)	21 (42)	0.587 ^χ2^
Cataract			
Cortical *n* (%)	54 (35.5)	50 (29.9)	0.288 ^χ2^
Nuclear *n* (%)	42 (27.6)	23 (13.8)	**0.002** ^χ2^
Subcapsular *n* (%)	11 (7.23)	20 (11.9)	0.154 ^χ2^

*^t^* Independent sample *t*-test, *^U^* Mann–Whitney U test, and ^χ2^ Chi-square test. Data are presented as mean ± standard deviation, median (IQR), or percentage (*n*, %), as appropriate. DM, diabetes mellitus; BMI, body mass index; HbA1c, hemoglobin A1c; CVD, cardiovascular disease; CKD, chronic kidney disease; DPN, diabetic polyneuropathy; IOP, intraocular pressure. Statistically significant differences, indicated by a *p*-value of <0.05, are highlighted in bold.

**Table 4:** Predictors for cataract occurrence in the entire study population.

Variable	SE	OR (95% CI)	*p*-Value
Age (years)	0.016	1.055 (0.023–0.084)	**<0.001**
DM duration (years)	0.024	1.132 (0.077–0.170)	**<0.001**
HbA1c (%)	0.118	1.296 (0.027–0.491)	**0.029**
CKD	0.303	2.688 (0.396–1.582)	**0.001**
CVD	0.285	1.089 (−0.473–0.643)	0.765
Dyslipidemia	0.272	1.367 (−0.220–0.846)	0.250
DPN	0.280	1.928 (0.107–1.206)	**0.019**
Hepatic steatosis	0.409	1.643 (−0.305, 1.297)	0.225

Abbreviations: SE, standard error; OR, odds ratio; CI, confidence interval; DM, diabetes mellitus; HbA1c, hemoglobin A1c; CKD, chronic kidney disease; CVD, cardiovascular disease; DPN, diabetic polyneuropathy. Statistically significant differences, indicated by a *p*-value of <0.05, are highlighted in bold.

**Table 5:** Comparison of demographic and clinical parameters for identifying patients with cataracts across the entire study population.

Variable	AUC	SE	95% CI	Sensitivity	Specificity	Cutoff	*p*-Value
Age	0.646	0.0335	0.580–0.711	46.7	77.1	>74	**<0.001**
Disease duration	0.702	0.0344	0.635–0.770	49.5	92.9	>14	**<0.001**
HbA1c	0.611	0.0328	0.546–0.675	35.9	80	>6.8	**<0.001**

Abbreviations: AUC, area under the curve; SE, standard error; CI, confidence interval; HbA1c, hemoglobin A1c. Statistically significant differences, indicated by a *p*-value < 0.05, are highlighted in bold.

**Table 6:** Comparison of potential risk factors across different types of cataracts in type 2 diabetes patients.

Confounding Factor	Group Factor	Nuclear Cataracts			Cortical Cataracts			Posterior Subcapsular Cataracts		
		OR	95% CI	*p*-Value	OR	95% CI	*p*-Value	OR	95% CI	*p*-Value
Dyslipidemia	HbA1c ≥ 7%	5.48	1.24–24.03	**0.01**	5.78	1.30–25.63	**0.01**	2.52	0.28–22.10	0.38
	HbA1c < 7%	0.18	0.04–0.80	**0.01**	0.17	0.03–0.76	**0.01**	0.39	0.04–3.46	0.38
CKD	HbA1c ≥ 7%	4.34	1.13–16.61	**0.02**	4.30	1.11–16.58	**0.02**	2.35	0.26–20.83	0.43
	HbA1c < 7%	0.23	0.06–0.87	**0.02**	0.23	0.06–0.89	**0.02**	0.42	0.04–3.75	0.43
CVD	HbA1c ≥ 7%	5.62	1.27–24.79	**0.01**	6.19	1.40–27.32	**0.006**	2.66	0.30–23.15	0.35
	HbA1c < 7%	0.17	0.04–0.78	**0.01**	0.16	0.03–0.71	**0.006**	0.37	0.04–3.25	0.35
Smoker	HbA1c ≥ 7%	5.17	1.18–22.62	**0.01**	5.55	1.26–24.43	**0.01**	2.51	0.28–21.79	0.38
	HbA1c < 7%	0.19	0.04–0.84	**0.01**	0.17	0.04–0.79	**0.01**	0.39	0.04–3.45	0.38
BMI *	HbA1c ≥ 7%	2.55	0.87–7.41	0.08	2.81	0.95–8.28	0.05	1.16	0.29–4.62	0.83
	HbA1c < 7%	0.39	0.13–1.13	0.08	0.35	0.12–1.04	0.05	0.86	0.21–3.42	0.83
DR	HbA1c ≥ 7%	5.38	1.22–23.61	**0.01**	5.71	1.30–25.05	**0.009**	2.77	0.32–23.8	0.32
	HbA1c < 7%	0.18	0.04–0.81	**0.01**	0.17	0.03–0.76	**0.009**	0.35	0.04–3.08	0.32
DPN	HbA1c ≥ 7%	4.76	1.22–18.45	**0.01**	5.21	1.32–20.47	**0.01**	2.57	0.29–22.3	0.37
	HbA1c < 7%	0.21	0.05–0.81	**0.01**	0.19	0.04–0.75	**0.01**	0.38	0.04–3.36	0.37
Hepatic steatosis	HbA1c ≥ 7%	5.40	1.22–23.93	**0.01**	5.78	1.29–25.91	**0.01**	2.39	0.27–20.6	0.41
	HbA1c < 7%	0.18	0.04–0.81	**0.01**	0.17	0.03–0.77	**0.01**	0.41	0.04–3.61	0.41
Age **	HbA1c ≥ 7%	3.53	1.10–11.33	**0.02**	3.74	1.15–12.13	**0.01**	1.54	0.30–7.78	0.59
	HbA1c < 7%	0.28	0.08–0.90	**0.02**	0.26	0.08–0.86	**0.01**	0.64	0.12–3.25	0.59
Diabetes duration **	HbA1c ≥ 7%	3.60	1.02–12.59	**0.03**	4.47	1.25–16.01	**0.01**	1.38	0.26–7.14	0.69
	HbA1c < 7%	0.27	0.07–0.97	**0.03**	0.22	0.06–0.79	**0.01**	0.72	0.13–3.71	0.69
CKD	Retinopathy	1.37	0.51–3.69	0.52	3.90	1.54–9.92	**0.002**	15.79	1.77–140.3	**0.001**
CKD	CVD	1.30	0.48–3.47	0.59	3.48	1.37–8.78	**0.005**	15.99	1.82–139.7	**0.0009**

Cochran–Mantel–Haenszel odds ratio analysis. HbA1c, hemoglobin A1c; CKD, chronic kidney disease; CVD, cardiovascular disease; BMI, body mass index; DR, diabetic retinopathy; DPN, diabetic polyneuropathy. * BMI was tested as a class of nutritional status: underweight, normal weight, overweight, and obesity grades 1, 2, and 3. ** Age and diabetes duration were divided into four equal quantiles and tested for each group. Patients with a diabetes duration of a median 18 (min 15, max 45) years (quartile 4) and an HbA1c ≥ 7% had a 4.47 OR (95% CI: 1.15–113.11, *p* = 0.01), versus those with an HbA1c < 7% who had an 0.22 OR (95% CI: 0.008–0.86, *p* = 0.01) for developing cortical cataracts. Statistically significant differences, indicated by a *p*-value of <0.05, are highlighted in bold.

## Data Availability

All available data can be provided upon request from the corresponding author.
